# P01-025 – Decreased vitamin D levels in children with FMF

**DOI:** 10.1186/1546-0096-11-S1-A29

**Published:** 2013-11-08

**Authors:** B Makay, A Anık, G Çatlı, A Abacı, T Küme, E Böber, E Ünsal

**Affiliations:** 1 Pediatric Rheumatology, Dokuz Eylül University Hospital, İzmir, Turkey; 2 Pediatric Rheumatology, Dokuz Eylül University Hospital, İzmir, Turkey; 3 biochemistry, Dokuz Eylül University Hospital, İzmir, Turkey

## Introduction

Several recent studies have reported a link between vitamin D deficiency and certain chronic inflammatory disorders such as rheumatoid arthritis (RA), systemic lupus erythematosus (SLE) and Behçet’s disease.These recent findings have led to greater emphasis on treatment of vitamin D deficiency and vitamin D supplementation in rheumatological diseases. To our knowledge, vitamin D levels have not been previously investigated in children with FMF disease.

## Objectives

To determine the frequency of vitamin D deficiency in children with familial Mediterranean fever (FMF) and to investigate the factors associated with low vitamin D status.

## Methods

Forty-four patients with FMF and 39 age- and sex-matched healthy controls were enrolled in this study. Demographic data, disease duration, time to delay for diagnosis, FMF symptoms, disease severity score, MEFV mutation, dose and duration of colchicine therapy and compliance to treatment were recorded for each patient. Serum 25- hydroxyvitamin D levels were measured by original commercial kit based on Chemiluminescent Microparticle İmmunoassay (CMIA) principle.

## Results

The serum 25- hydroxyvitamin D levels were significantly lower in FMF patients than the healthy controls (12.9 ± 3.6 and 16.3 ± 5.5, respectively, p=0.001). The vitamin D level was similar in patients homozygous for M694V and other genotypes (11.8 ± 3.7 and 13.2 ± 3.6, respectively, p=0.21). There was a significant negative correlation between the duration and cumulative dose of colchicine use and vitamin D levels (r=-0.410, p=0.006 and r=-443, p=0.004, respectively). There was no correlation between vitamin D levels and C-reactive protein, white blood cell count, disease duration, disease severity score or age of the patient.

## Conclusion

The results of this study suggest that serum 25- hydroxyvitamin D levels are decreased in children with FMF. Duration of colchicine use and cumulative colchicine dose appear to effect vitamin D levels negatively.

## Disclosure of interest

None declared.

